# Happiness in texting times

**DOI:** 10.3389/fpsyg.2015.01436

**Published:** 2015-09-25

**Authors:** David Hevey, Karen Hand, Malcolm MacLachlan

**Affiliations:** ^1^School of Psychology, Trinity College Dublin, Dublin 2Ireland; ^2^Centre for Global Health, Trinity College Dublin, Dublin 2Ireland

**Keywords:** happiness, well-being, longitudinal, population, SMS

## Abstract

Assessing national levels of happiness has become an important research and policy issue in recent years. We examined happiness and satisfaction in Ireland using phone text messaging to collect large-scale longitudinal data from 3,093 members of the general Irish population. For six consecutive weeks, participants’ happiness and satisfaction levels were assessed. For four consecutive weeks (weeks 2–5) a different random third of the sample got feedback on the previous week’s mean happiness and satisfaction ratings. Text messaging proved a feasible means of assessing happiness and satisfaction, with almost three quarters (73%) of participants completing all assessments. Those who received feedback on the previous week’s mean ratings were eight times more likely to complete the subsequent assessments than those not receiving feedback. Providing such feedback data on mean levels of happiness and satisfaction did not systematically bias subsequent ratings either toward or away from these normative anchors. Texting is a simple and effective means to collect population level happiness and satisfaction data.

## Introduction

The study of happiness and life satisfaction at an individual and societal level has gained importance not only for psychological science (Diener and Chan, 2011; Forgeard et al., 2011; Kahneman, 2011), but also for social and economic policy (e.g., Organization for Economic Cooperation and Development [Oecd], 2001, 2011). Consequently, the optimal methods for the routine assessment of these factors in real-life situations require examination. Previous research examining happiness and satisfaction has typically assessed such variables using questionnaires (Diener et al., 1985; Fordyce, 1988) administered face to face with respondents. In addition, the day reconstruction method (DRM) has also been used to retrospectively measure the frequency and intensity of positive and negative emotions over a period of 24 h (Kahneman et al., 2004). Relatedly, experience sampling methods (ESM; Csikszentmihalyi et al., 1977; Scollon et al., 2007) use electronic devices (e.g., pager and mobile phone) to signal respondents during the day to answer questions regarding their current feelings; such studies typically last 1–2 weeks (Reis and Gable, 2000). ESM overcomes the memory biases associated with retrospective methods, such as the DRM, by asking respondents to describe their current psychological state when answering the questions. Furthermore, by collecting longitudinal data from a sample with repeated measures ESM facilitates detailed examination of within-person fluctuations in happiness and satisfaction. A number of previous studies (Emmons, 1986; Freeman et al., 1986; Moneta et al., 2001) have used ESM to measure participant mood states over time but such data collection was done using written forms and web-forms. Although collecting such data was initially very costly and time-consuming, ESM can now be easily implemented using mobile phone technology.

Short message service (SMS) texting provides the potential to routinely collect data from large-scale representative samples from the population at little cost. As noted by Miller (2012), due to their ubiquity, phones can facilitate the gathering of ecologically valid data on the experiences of millions of people in a convenient and unobtrusive manner. The widespread adoption of mobile technology by populations opens up new research programs and provides enhanced understanding of the everyday experience of individuals (Reiss et al., 2014). For the past two decades researchers have used mobile electronic devices to collect research data and although SMS texting has been used by psychologists in time-based studies to look at mood variations (Killingsworth and Gilbert, 2010), the method has not been used to measure the happiness and satisfaction of a sample of people over time.

In addition to collecting data, texting can also be used to provide information to research participants. For example, participants could receive feedback on the average levels of happiness and satisfaction for the sample. Such information may increase participants’ motivation to continue to provide data, which may overcome well-noted problems arising from high levels of attrition in longitudinal research (Patel et al., 2003). Previous studies have established that providing personalized feedback can successfully reduce attrition rates in longitudinal interventions and surveys. Marcus et al. (2007) found that providing personalized feedback to participants in a randomized controlled trial (RCT) to enhance physical activity maintained a high level of participation. Bewick et al. (2008) reported that providing personalized feedback on alcohol consumption was effective for retaining participants in the research. Fumagalli et al. (2013) found that providing salient feedback was more successful in reducing survey attrition rates than financial incentives. Providing participants with personalized information that is highly salient to the individual can maintain high levels of participation rates; however, the effect of giving non-personalized information is unclear. Research indicates that provision of even minimal feedback can give a task purpose, which consequently increases motivation of participants to perform at levels above those for individuals not receiving feedback (Ariely et al., 2009). Furthermore, while general feedback may enhance motivation, awareness of the average level of happiness and satisfaction among a general sample might provide a reference point for comparison that influences the individuals’ subsequent ratings.

Social comparison theory states that when objective standards do not exist, people compare their beliefs, achievements and abilities with others’ beliefs, achievements and abilities (Festinger, 1954). The referent group for such comparisons varies across time and context; however, research indicates that social comparisons are associated with levels of happiness (Gutek et al., 1983; Emmons and Diener, 1985; Diener and Shigehiro, 2005). Once presented with the mean level for others, participants may be motivated to increase their own reported levels of happiness and satisfaction to be “better-than-average”, a pervasive self-enhancement bias noted in the literature (Alicke and Govorun, 2005). Alternatively, individuals may use such values as anchors that may subsequently bias their subsequent happiness and satisfaction ratings toward the mean values (Furham and Boo, 2011).

In summary, the study contributes to the literature by using SMS to collect weekly data on happiness and satisfaction in a large national sample. The provision of general feedback offers a novel distinction between the present study and the previous studies, which have used personalized feedback typically related to a salient health behavior. In addition, the effects of receiving feedback on the average level of happiness and satisfaction from the previous week on (i) participant retention, and (ii) subsequent levels of happiness and satisfaction were assessed.

## Materials and Methods

### Participants

Three thousand ninety-three people were recruited through national media (i.e., write-ups in national newspapers, discussion on national TV and radio, and posters in prominent and highly visible city centre locations) publicity for a study investigating happiness in Ireland. Participants sent a text to register their interest with the researchers; all personal data were anonymous, participation was free and all participants’ return texts were free.

### Procedure

Following receipt of ethical approval from the University’s School of Psychology Research Ethics Committee, the study ran over 6 weeks during summer: each week questions were sent via SMS. Once the first question was answered, the response was acknowledged, and the second question was sent. Each week, the SMS was sent on a Thursday and from the second week onwards, after completing that week’s questions, one-third of the participants were randomly selected to get feedback on the previous week’s average results for all participants. For example, at the end of week 2 those in the feedback condition received the following message:

“*Thanks for taking the time to answer this week’s questions. We will contact you next week for more information. In week 1, the average happiness score for all respondents was 7.04 and the average life satisfaction score was 6.71.”*

After providing their responses on week 2, the Control Group were told

“Thanks for taking the time to answer this week’s questions. We will contact you next week for more information.”

For 4 weeks (weeks 2–5) a different random third of the sample got feedback on the previous week’s mean levels of happiness and satisfaction for all participants; consequently, over the course of the study 2,529 participants received feedback (965 received feedback once, 963 received it twice, 503 received it three times, and 98 received it four times) and 564 never received feedback.

### Measures

Participants’ gender and age were recorded the first week, and their current happiness (“How happy do you feel now?”) and satisfaction (“How satisfied with life in general do you feel?”) levels at each time point were assessed using separate 10-point rating scales (anchored by 1 = “not at all” and 10 = “very much”). Such single-item subjective scales have been validated within happiness and well-being research (Abdel-Khalek, 2006).

### Analysis

Data were initially screened to examine univariate and multivariate normality and outliers using a combination of graphical (e.g., box plots, histograms, and residual plots) and statistical (e.g., Mahalanobis distance) methods. Feedback effects over time were evaluated using multilevel modeling (MLM), which is appropriate to the evaluation of data that have a hierarchical structure (i.e., up to six assessment times nested within each of the participants, who were nested in feedback groups) because it is able to account for variation both within and between individuals^1^. MLM analyses were conducted using SPSS, estimating the variance components using restricted maximum likelihood. Models included the predictors time (T1, T2, T3, T4, T5, and T6), group (feedback vs. no feedback), and the time X group interaction. To further examine the effects of the getting feedback on the sample’s mean happiness and satisfaction levels on each participant’s subsequent ratings, the difference between these means and the individual ratings were calculated such that positive numbers meant that the individual had a higher level of happiness/satisfaction than the sample mean, zero reflected no difference between the individual’s ratings and the sample mean, and a negative number meant that the individual scored lower than the sample mean.

In addition, a series of *F* tests examining the variance ratios at each of the weeks where feedback could have had an effect were conducted to determine if provision of feedback resulted in less variance around the mean values^1^. Comparisons between groups were performed using *t*-tests, with Cohen’s *d* indexing effect size. Both linear and non-linear models were examined to best represent the relationship between age, happiness and satisfaction. Relationships between categorical variables were assessed using χ^2^ and odds ratios. Given the large sample size, statistical significance for all analyses was set at *p* < 0.01.

## Results

The sample was predominantly female (76%) and the average age was 45.3 years (*SD* = 13.4, range 18–84). One half of the sample was aged between 40 and 59 years; over half were parents (59%). The weekly ratings of happiness and satisfaction are presented in **Table 1**.

**Table 1:** *M* (*SD*) for happiness and satisfaction ratings for the 6 weeks.

	Week 1	Week 2	Week 3	Week 4	Week 5	Week 6
Happiness *M* (*SD*)	7.04 (1.9)	6.91 (1.99)	6.61 (1.93)	6.68 (1.92)	6.66 (1.96)	6.77 (1.95)
Satisfaction *M* (*SD*)	6.71 (1.80)	6.78 (1.81)	6.64 (1.77)	6.58 (1.83)	6.66 (1.85)	6.62 (1.86)

**FIGURE 1:**
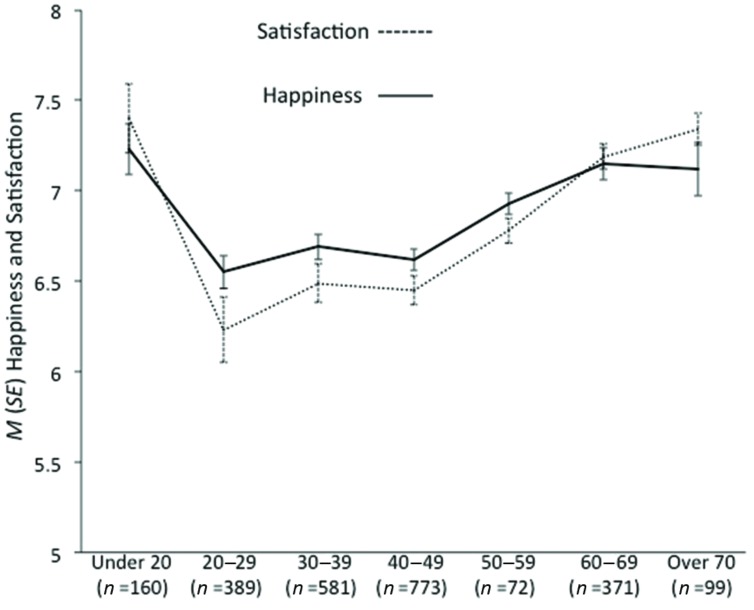
***M (SE)* happiness and satisfaction levels by age category**.

Over the course of the 6 weeks the mean level of happiness was 6.80 (*SD* = 1.38) and was 6.66 (*SD* = 1.54) for satisfaction. There were no significant gender differences for either average happiness [*M*_female_ = 6.79 vs. *M*_male_ = 6.82, *t*(2,011) = -0.39, *p* = 0.66, *d* = 0.01] or satisfaction [*M*_female_ = 6.69 vs. *M*_male_ = 6.87, *t*(1,806) = -0.001, *p* = 0.93; *d* < 0.01]. There was a significant non-linear relationship between age and both happiness (Spearman’s ρ = 0.16, *p* < 0.001) and satisfaction (Spearman’s ρ = 0.16, *p* < 0.001); the mean levels for each variable across different age categories displayed a U-shaped curve (**Figure 1**).

### Effects of Feedback

Over the course of the study 825 of the initial participants dropped out: there was a significantly higher level of drop out in the group that never received feedback (64% dropped out) than in the group who received feedback (17% dropped out), χ^2^ (1, *N* = 3,093) = 486.91, *p* < 0.001. The relationship between frequency of receiving feedback and drop out is presented in **Table 2**. There was a significant linear association between levels of feedback and drop-out levels, χ^2^ (4, *N* = 3,093) = 466.45, *p* < 0.001. On a week by week basis the computer-generated random sample who received feedback on the previous week’s mean levels of happiness and satisfaction were more likely to provide data at each time point; in addition, the more feedback one received the less likely one was to drop out.

**Table 2:** Relationship between frequency of feedback received and drop out.

Receive feedback….	Drop out *n* (%)	Complete study *n* (%)
Never	360 (64)	204 (36)
Once	262 (27)	703 (73)
Twice	161 (17)	802 (83)
Three times	38 (8)	465 (92)
Four times	4 (4)	94 (96)

Aggregating over the course of the study, those who received any feedback were eight times (OR = 7.83, 95% CI = 6.41–9.56, *z* = 20.27, *p* < 0.001) more likely to provide data every week than those who never received feedback.

The MLM analysis in relation to happiness showed that there was no significant main effect for feedback [*F*(1,12821.45) = 0.346, *p* = 0.56] or interaction effect of feedback by time [*F*(1,12533.05) = 1.88, *p* = 0.17]; however, there was a main effect of time [*F*(1,10405.61) = 37.77, *p* < 0.001]. Mean levels of happiness at week 1 were significantly higher than the means for weeks 3, 4, 5, and 6. Week 2’s mean was similarly significantly higher than the means for weeks 3, 4, 5, and 6. Week 6’s mean was significantly higher than week 3’s mean. No other comparisons were significant.

In relation to satisfaction, there was no significant main effect of feedback [*F*(1,10324.58) = 1.80, *p* = 0.18] or interaction effect of feedback by time [*F*(1,10214.05) = 1.71, *p* = 0.19]; however, there was a main effect of time [*F*(1,10600.86) = 5.21, *p* < 0.05]. Mean levels of satisfaction at week 1 were significantly higher than weeks 4 and 6. Week 2’s mean was higher than the means at weeks 3, 4, 5, and 6. No other comparisons were significant.

**Table 3** presents the comparisons between the groups who got feedback with those who received no feedback. As the first feedback was provided after participants had completed their week 2 ratings, the first ratings that feedback could have influenced were on week 3. For example, on week 2 those who received feedback were informed the average level of happiness was 7.04; this value served as the comparison for the week 3 ratings. **Table 3** shows that those who got feedback on week 2 had a mean rating of happiness on week 3 that was 0.41 units below 7.04, whereas those who did not get any feedback on week 2 had a mean rating of happiness on week 3 that was 0.43 units below 7.04. There was no significant difference between the feedback and no feedback groups on the mean difference of their week 3 ratings (-0.41 vs. -0.43) to the comparison mean (7.04). These comparisons were conducted for each of the 4 weeks where feedback could have an effect on the subsequent ratings. Examination of the differences between the individuals’ ratings and the comparison mean showed that on a week by week basis there were no significant differences between the groups at the *a priori* selected 0.01 level; however, it should be noted that at week 5 the differences between the groups in relation to Happiness [*t*(2,682) = -2.35, *p* = 0.019] and Satisfaction [*t*(2,592) = -2.23, *p* = 0.026] would be considered significant at the 0.05 level^1^. The feedback group’s means were lower than the previous week’s levels of happiness and satisfaction, whereas the no feedback group’s means were above the previous week’s levels. Notably, the effect sizes for the differences between the ratings were generally very small to trivial, with the largest difference emerging at week 5.

**Table 3:** Differences in happiness and satisfaction ratings between feedback and no feedback group for each week relative the feedback values from the previous week.

	Feedback	No feedback	*t*	*d*	Variance ratio
	*M* (*SD*)	*M* (*SD*)			
**Week 3 rating**					
Comparison happiness					
Week 2, *M* = 7.04	-0.41 (1.92)	-0.43 (1.94)	0.27	0.01	0.98
Comparison satisfaction					
Week 2, *M* = 6.71	-0.05 (1.74)	-0.07 (1.78)	0.25	0.01	0.96
**Week 4 rating**					
Comparison happiness					
Week 3, *M* = 6.91	-0.18 (1.88)	-0.24 (1.95)	0.81	0.03	0.93
Comparison satisfaction					
Week 3, *M* = 6.78	-0.17 (1.77)	-0.18 (1.89)	0.13	<0.01	0.88
**Week 5 rating**					
Comparison happiness					
Week 4, *M* = 6.71	-0.03 (1.99)	0.08 (1.94)	-2.35	-0.06	1.05
Comparison satisfaction					
Week 4, *M* = 6.67	-0.05 (1.90)	0.08 (1.82)	-2.23	-0.07	1.09
**Week 6 rating**					
Comparison happiness					
Week 5, *M* = 6.66	0.04 (1.96)	0.14 (1.95)	-1.35	-0.05	1.01
Comparison satisfaction					
Week 5, *M* = 6.66	0.05 (1.85)	0.04 (1.88)	0.08	<0.01	0.97

Over the course of the study the means for the feedback group were not significantly closer to or further away from the comparison means than the means for the no feedback group. Comparison of the variance ratios (ranging from 0.88 to 1.09) showed that there were no significant differences (0.07 < *p* < 0.99) between the groups in relation to the variances at each assessment point for either happiness or satisfaction ratings. Provision of feedback did not result in less variance around the mean compared to not having received feedback.

In addition, there was no difference in overall average happiness [*F*(4,2228) = 0.98, *p* > 0.05] or satisfaction [*F*(4,1941) = 1.15, *p* > 0.05] between the sample classified according to the amount of times (0–4) they received feedback; a comparison of those who received feedback at least once vs. those who never received feedback also revealed no difference in overall happiness [*t*(2,227) = -1.66, *p* > 0.05] or satisfaction [*t*(1,940) = -0.94, *p* > 0.05]. Furthermore, comparison of those who never got feedback during the study (*n* = 564) vs. those who got feedback on their happiness and satisfaction levels showed no difference between them in their weekly happiness (0.07 < *p* < 0.99) or satisfaction (0.08 < *p* < 0.81) levels.

## Discussion

Text messaging provided data on national levels of happiness and satisfaction for a large sample in a novel longitudinal study. The present results are similar to those reported using traditional pen-and-paper methods to assess happiness and satisfaction; national levels of happiness and satisfaction are in line with the values reported using standard survey methodology (Organization for Economic Cooperation and Development [Oecd], 2011). In addition, the analysis revealed a U-shaped relationship between age and both happiness and satisfaction. Such a finding is congruent with recent research on happiness; for example, Blanchflower and Oswald (2008) used a variety of data sets that could distinguish age effects from cohort effects to suggest that the U-shaped structure of the curve is broadly consistent across countries throughout the globe. Overall, the present findings are consistent with those reported using standard methods and provide evidence for the validity of the method to obtain meaningful psychological data in an efficient manner using mobile phone technology.

Those who received feedback were more likely to provide complete data across repeated assessments; this pattern is consistent with Ariely et al.’s (2009) argument that feedback provides ‘minimal meaning’ to a task and enhances motivation regarding task performance. Those who received feedback did not remain in the study due to feeling happier or more satisfied in general. Provision of summary data on happiness and satisfaction did not systematically bias subsequent ratings in relation to sample mean normative anchors; this finding, if replicated, indicates that use of such minimal feedback does not distort individual responses. However, it should be noted that although the effect sizes were small (-0.07 < *d* < 0.03), the differences at week 5 would be considered statistically significant using a less stringent value of 0.05. Clearly, addition research is required to determine whether such differences represent systematic and substantive effects of generalized feedback.

Furthermore, it must be acknowledged that participants may not have perceived the generalized feedback (i.e., the mean ratings) as being salient; given the diversity of the sample (e.g., in terms of age range) used to calculate the means participants may not have regarded the mean value as being relevant. For example, the significant association between age and happiness/satisfaction ratings (**Figure 1**) indicates that different age groups may have different normative levels of happiness and satisfaction. Given such an association, it is plausible that an effect of feedback could be significant if such feedback was tailored based on the participant’s demographic profile (e.g., age or gender). Consequently, although general feedback had little effect on ratings, it is possible that salient personalized feedback would serve as an anchor to subsequently bias individuals’ subsequent happiness and satisfaction ratings toward the mean values (Furham and Boo, 2011). Such a possibility requires empirical investigation.

In comparison to standard longitudinal research methods (Patel et al., 2003), the relatively low drop-out rate, particularly among those receiving generalized feedback, is a novel finding that highlights the value of texting as a simple and effective means to provide longitudinal data on psychological phenomena. Previous research had documented that personally salient feedback impacted upon participation; the present study adds to this literature by providing evidence that generalized feedback may have a similar effect. This study also indicates one of the means by which texting data can contribute to producing ‘Big Data’ and addressing macropsychology questions (MacLachlan, 2014).

### Limitations

It must be acknowledged that all measures were succinct given the methodological constraints of SMS. In addition, the sample self-selected, which may limit generalizability, and of note, the sample can be characterized as being predominantly middle-aged women. Replication of the study using a broader range of questions on socio-demographic (e.g., education, ethnicity, wealth, and mobile phone use), and psychological factors with different samples is required.

## Conclusion

The present study provides novel data to indicate that collecting longitudinal happiness and satisfaction data from a large sample is feasible using SMS text messaging. Provision of summary feedback to participants may diminish attrition rates without biasing their individual responses relative to potential normative anchors or social comparison targets.

## Conflict of Interest Statement

The authors declare that the research was conducted in the absence of any commercial or financial relationships that could be construed as a potential conflict of interest.

## Footnotes

1 We wish to thank anonymous reviewers for raising these issues.
